# A Novel, Apparently Silent Variant in *MFSD8* Causes Neuronal Ceroid Lipofuscinosis with Marked Intrafamilial Variability

**DOI:** 10.3390/ijms23042271

**Published:** 2022-02-18

**Authors:** Milda Reith, Lena Zeltner, Karin Schäferhoff, Dennis Witt, Theresia Zuleger, Tobias B. Haack, Antje Bornemann, Michael Alber, Susanne Ruf, Ludger Schoels, Katarina Stingl, Nicole Weisschuh

**Affiliations:** 1 Centre for Ophthalmology, University Eye Hospital, University of Tübingen, 72076 Tübingen, Germany; milda.reith@med.uni-tuebingen.de (M.R.); katarina.stingl@med.uni-tuebingen.de (K.S.); 2 Department of Neurodegenerative Disease, Hertie-Institute for Clinical Brain Research and Center for Neurology, University of Tübingen, 72076 Tübingen, Germany; lena.zeltner@med.uni-tuebingen.de (L.Z.); ludger.schoels@uni-tuebingen.de (L.S.); 3 Centre for Rare Diseases, University of Tübingen, 72076 Tübingen, Germany; tobias.haack@med.uni-tuebingen.de; 4 Institute of Medical Genetics and Applied Genomics, University of Tübingen, 72076 Tübingen, Germany; karin.schaeferhoff@med.uni-tuebingen.de (K.S.); dennis.witt@med.uni-tuebingen.de (D.W.); theresia.zuleger@med.uni-tuebingen.de (T.Z.); 5 Department of Neuropathology, University Hospital Tübingen, University of Tübingen, 72076 Tübingen, Germany; antje.bornemann@med.uni-tuebingen.de; 6 Department of Neuropaediatrics, Developmental Neurology, Social Paediatrics, University Children’s Hospital, 72076 Tübingen, Germany; michael.alber@med.uni-tuebingen.de (M.A.); susanne.ruf@med.uni-tuebingen.de (S.R.); 7 Centre for Ophthalmology, Institute for Ophthalmic Research, University of Tübingen, 72076 Tübingen, Germany

**Keywords:** CLN7, *MFSD8*, synonymous substitution, mis-splicing, exon skipping, functional studies, inherited retinal disease, retinopathy, neuronal ceroid lipofuscinosis, genome sequencing

## Abstract

Variants in *MFSD8* can cause neuronal ceroid lipofuscinoses (NCLs) as well as nonsyndromic retinopathy. The mutation spectrum includes mainly missense and stop variants, but splice sites and frameshift variants have also been reported. To date, apparently synonymous substitutions have not been shown to cause *MFSD8*-associated diseases. We report two closely related subjects from a consanguineous Turkish family who presented classical features of NCLs but demonstrated marked intrafamilial variability in age at the onset and severity of symptoms. In fact, the difference in the onset of first neurologic symptoms was 15 years and that of ophthalmologic symptoms was 12 years. One subject presented an intellectual disability and a considerable cerebellar ataxia syndrome, while the other subject showed no intellectual disability and only a mild atactic syndrome. The diagnostic genetic testing of both subjects based on genome sequencing prioritized a novel, apparently synonymous variant in *MFSD8*, which was found in homozygosity in both subjects. The variant was not located within an integral part of the splice site consensus sequences. However, the bioinformatic analyses suggested that the mutant allele is more likely to cause exon skipping due to an altered ratio of exonic splice enhancer and silencer motifs. Exon skipping was confirmed in vitro by minigene assays and in vivo by RNA analysis from patient lymphocytes. The mutant transcript is predicted to result in a frameshift and, if translated, in a truncated protein. Synonymous variants are often given a low priority in genetic diagnostics because of their expected lack of functional impact. This study highlights the importance of investigating the impact of synonymous variants on splicing.

## 1. Introduction

The neuronal ceroid lipofuscinoses (NCLs) are a group of mainly autosomal, recessively inherited neurodegenerative disorders. They belong to the lysosomal storage diseases and are characterized by an accumulation of autofluorescent lipopigments in a range of different cell types and by a loss of neuronal cells in brain and retina [1]. Historically, the different forms of NCLs were classified according to their ages of onset and clinical symptoms, but the most recent classification system is primarily based on their underlying genetic cause [2]. Clinically, the presence, onset, and sequence of symptoms vary widely between the different forms but generally include seizures, retinopathy, and progressive decline in cognitive and motor functions [3]. The underlying disease mechanisms of NCLs are poorly understood [4,5,6]. For a long time, neurodegeneration in NCLs was thought to be caused by the toxicity of accumulated storage material. However, the pathology might be more complex, as the amount of storage material and neuronal loss are not necessarily strongly correlated [4,7].

Currently, 14 forms of NCLs are known. The numbering of the individual forms (CLN1−CLN14) is based on the historical order of discovery. CLN7 (MIM *#610951)* is caused by biallelic pathogenic variants in *MFSD8* [8]. Based on sequence homology, the CLN7 protein belongs to the major facilitator superfamily (MFS) of secondary active transporters. MFS transporters translocate small solutes across membranes in response to chemiosmotic ion gradients [9]. To date, the substrate and function of the CLN7 protein are unknown. *MFSD8* is expressed ubiquitously, with the highest expression levels in the cerebral cortex and cerebellum, as was shown in mice and rats [10,11,12]. Patients with biallelic variants in *MFSD8* usually present classical NCL symptoms such as seizures, visual loss, mental regression, and ataxia [8,13,14,15,16,17], but cases with isolated retinopathy have been reported as well [18,19,20,21,22,23]. The nonsyndromic manifestation has been attributed to the residual expression of hypomorphic *MFSD8* variants [18]. As of January 2022, the Human Gene Mutation Database (HGMD) [24] has listed 67 disease-causing variants in *MFSD8*. The mutation spectrum includes missense, nonsense, and splice site variants, as well as small insertions and deletions and one large deletion. Among the 15 splice alterations, 14 are located in introns, most of which are at the canonical splice sites. The only exonic variant reported to date to have an effect on splicing is a substitution of the last nucleotide of exon 11, which changes the highly conserved guanine at that position [25] to cytosine. The c.1102G > C variant is not only predicted to cause an amino acid substitution (p.(D386H)), but was also shown to promote exon skipping [18]. To the best of our knowledge, no pathogenic silent substitution has been reported in *MFSD8* to date.

In this study, we report a novel exonic variant in *MFSD8* detected in homozygosity in two closely related subjects with clinical features of NCL. This variant is silent at the translational level but affects splicing. Our study emphasizes the importance of analyzing synonymous substitutions that at first glance appear to be benign.

## 2. Results

### 2.1. Clinical Phenotypes

#### 2.1.1. Patient A

The male subject was 16 years old at the initial presentation to our department. The follow-up period was three years. According to his father, he showed the first symptoms at the age of four years, when speech regression was noticed. At the age of 10, he experienced blurred vision and difficulties in reading and was diagnosed with hereditary retinal dystrophy. At the same time, cognitive problems and gait disturbance were observed. After graduating from an elementary school, he attended a special school for the visually impaired. One year after the onset of the ophthalmological symptoms, the first epileptic seizure (focal to bilateral tonic−clonic) occurred. The epilepsy was successfully treated for three years with sultiam. Between the ages of 15 and 18 years, frequent seizures occurred during therapy with oxcarbazepine and valproic acid. The therapy was changed to levetiracetam, and the patient was seizure-free for at least 18 months.

**Figure 1:**
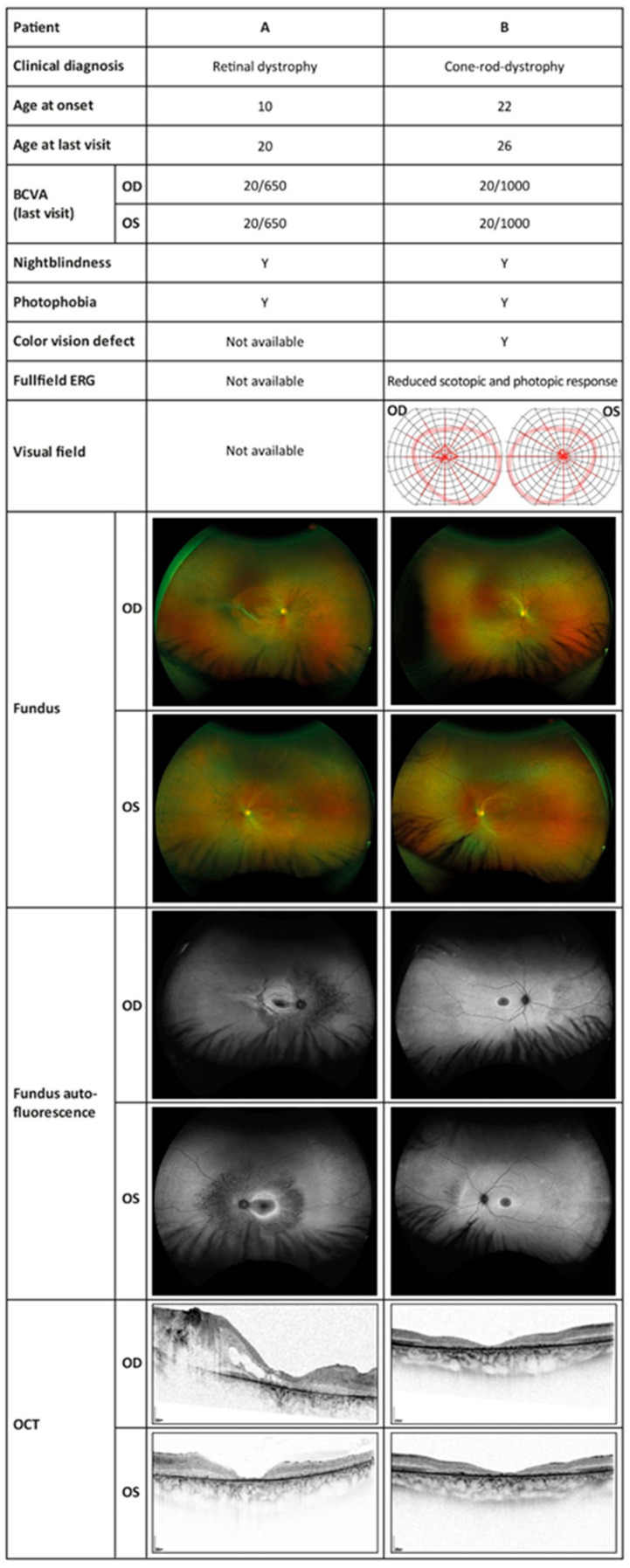
Ophthalmologic examinations showing the typical findings of retinal dystrophy in both subjects. BCVA, best corrected visual acuity; OD, right eye; OS, left eye; Y, yes; ERG, electroretinography; OCT, optical coherence tomography.

By age 16, the best corrected visual acuity (BCVA) was 20/320 in the right eye and 20/500 in the left eye. Optical coherence tomography (OCT) showed central and parafoveal loss of the photoreceptors as well as epiretinal gliosis in both eyes. Kinetic perimetry delivered unreliable results due to lacking compliance. Due to cognitive impairment and epilepsy, no electrophysiological examination was possible throughout the follow-ups. By age 18, OCT revealed a progressive loss of photoreceptors. The right eye developed a tractive membrane, and the inferior vascular arc caused a separation of the retinal layers with pseudocysts. In view of the poor prognosis, no surgical measures were taken. By age 19, visual acuity in both eyes had further decreased to 20/650, while the morphological findings in the OCT as well as in the fundus autofluorescence (FAF) had been stable. FAF showed a hypo-fluorescent fovea with hyper-fluorescent demarcation and a granular hypo-fluorescent pattern outside the vascular arc. Fundoscopy revealed retinal tearing in the right eye as well as atrophy in the macula with bone spiculae in the mid-peripheries of both eyes. A summary of ophthalmological findings is shown in Figure 1.

**Figure 2:**
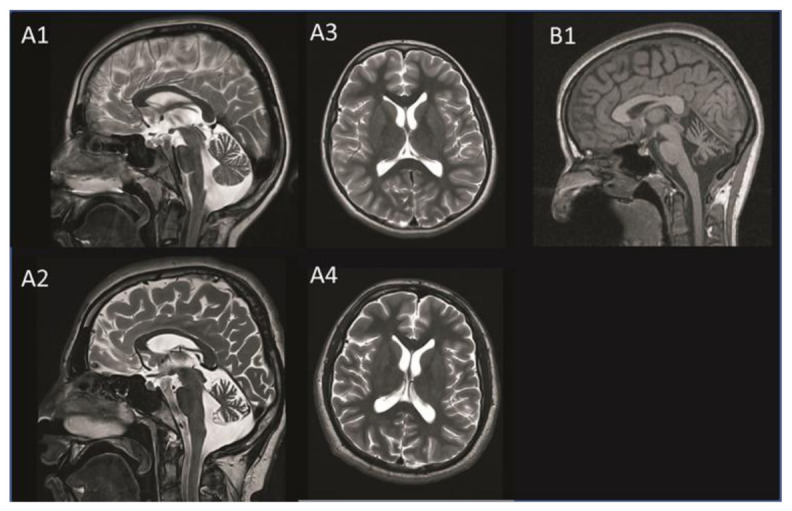
MRI images of patient A taken at age 11 (**A1** + **A3**) and age 16 (**A2** + **A4**) and those of patient B taken at age 20 (**B1**). Progressive cerebellar atrophy is visible on the T2-weighted saggital MRI image of patient A (**A1** + **A2**). The transversal MRI image shows progressive mild cerebral atrophy without white matter lesions in patient A (**A3** + **A4**). Cerebellar atrophy is visible on the T1-weighted saggital MRI image in patient B (**B1**). MRI, magnetic resonance imaging.

The neurological examination at the age of 19 confirmed a cognitive decline with amnestic deficits, comprehension problems for complex contents, and psychomotor deterioration. He showed a cerebellar syndrome with moderate dysarthria, saccadic gaze movements, and atactic gait. Walk without walking aids was safely possible, and tandem-walk was performed with uncertainty due to ataxia. The scale for the assessment and rating of ataxia scored 6/40 points. Cerebral magnetic resonance imaging (cMRI) showed progressive cerebellar atrophy and mild cerebral atrophy at the age of 16. Incidentally, a tumor of the pituitary gland (Rathke’s pouch) was found (Figure 2). The electron microscopic findings from a skin biopsy taken at age 16 were unremarkable and showed no evidence of lysosomal accumulation (data not shown).

#### 2.1.2. Patient B

The female subject was 23 years old at the initial presentation to our department. She first noticed blurred vision and reading difficulties at age 22, after which she was diagnosed with cone dystrophy. Apart from a febrile seizure at the age of two, her development in early childhood was normal. She graduated from high school and completed an apprenticeship. Since the age of 19, she had frequent bilateral tonic−clonic seizures and was diagnosed with epilepsy. At the age of 26, she reported frequent myoclonus of the extremities.

At the first examination in our department, she was 23 years old, and her visual acuity was 20/400 in both eyes. Kinetic perimetry showed normal borders with small central scotomas in static perimetry in both eyes. Electroretinography (ERG) of both eyes showed a significantly decreased photopic response and a near-normal scotopic response. In OCT, a central degeneration of the outer retinal layers without macular edema on both eyes was visible. The 30° FAF revealed a hypo-fluorescent macula with a hyper-fluorescent ring. After the three-year follow-up, general well-being was reduced, and she reported a recent epileptic seizure and a general weakness. The BCVA had deteriorated to 20/1000 in both eyes. Static perimetry showed a significant progression of the central scotomas in both eyes as well as a concentric constriction up to 2–10° in the left eye and 2–20° in the right eye in the kinetic perimetry. However, this finding might have been influenced by poor general health at the time of examination. The progression was reflected morphologically in the OCT with a slight progression in the photoreceptor atrophy. The changes in the FAF had progressed as could be seen by an expansion of the central macular atrophic area surrounded by a hyper-autofluorescent ring, as well as some granular peripheral hypo- and hyper-fluorescent areas in the nasal peripheral retina. The three-year-follow-up indicated a progression to cone-rod dystrophy. A summary of ophthalmological findings is shown in Figure 1.

The neurological examination at age 26 revealed ataxia of the extremities and mild unsteadiness in tandem walking. Oculomotor function and speech were normal, and there was no obvious mental retardation. No myoclonus was noted in the examination but was reported by the patient. She scored 4/40 points in the scale for the Assessment and Rating of Ataxia (SARA). The cMRI revealed cerebellar atrophy including the vermis cerebelli and cerebellar hemispheres (Figure 2). The electron microscopy image of a skin biopsy revealed vacuoled epithelial cells, compatible with lysosomes filled with normal lipofuscine (data not shown). Table 1 compares the clinical findings of both subjects.

### 2.2. Genetic Testing and Bioinformatic Analysis

**Figure 3:**
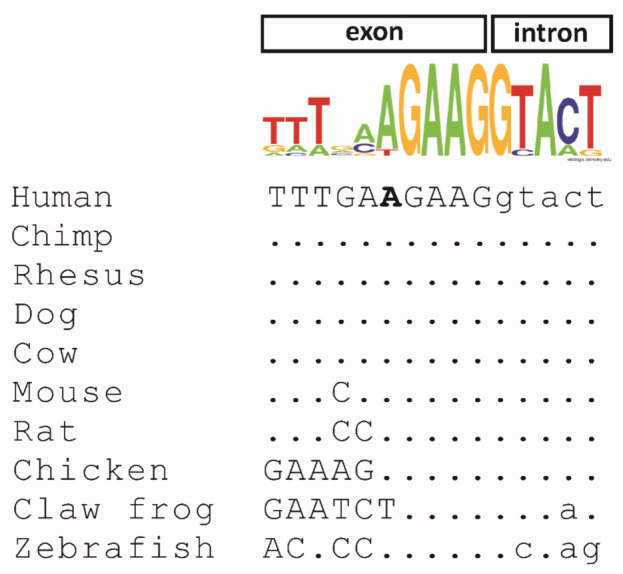
Evolutionary conservation of the c.750A nucleotide variant in 10 orthologous vertebrate sequences. Exonic nucleotides are shown in capital letters, and intronic nucleotides are indicated in lowercase letters. The c.750A nucleotide in the human sequence is shown in bold. RefSeq accession numbers are as follows: *Homo sapiens* (NM_152778.4), *Pan troglodytes* (XM_009448261.2), *Macaca mulatta* (XM_015139188.2), *Canis lupus* (XM_533294.7), *Bos taurus* (NM_001205823.1), *Mus musculus* (NM_028140.5), *Rattus norvegicus* (NM_001393796.1), *Gallus gallus* (XM_004941007.3), *Xenopus tropicalis* (XM_002932042.4), and *Danio rerio* (NM_001045048.1). The consensus sequence above the nucleotide alignment was created using Weblogo (logo@compbio.berkeley.edu). The height of each letter is proportional to the frequency of the corresponding nucleotide in the 10 analyzed species at the given position.

Both subjects in this study underwent diagnostic genetic testing by genome sequencing. The average coverages on target were 40x (98.69%; ≥20) for patient A and 48x (99.91%; ≥20) for patient B. Variants were prioritized that were rare (minor allele frequency: <0.01) and in known retinal disease genes (including syndromic disorders with retinal involvement). The only putative pathogenic variant identified was a single nucleotide substitution in the *MFSD8* gene (NM_152778.3: c.750A > G/p.E250E) that was present in a homozygous state in both patients. The variant was absent from different public databases, namely HGMD [24], dbSNP [26], ClinVar [27], the Genome Aggregation database (gnomAD) [28], and 1000 Genomes [29]. The respective nucleotide is located close to an exon−intron border in all vertebrates. In general, the outer regions of exons (i.e., close to the intron borders) show a significantly higher degree of evolutionary conservation and a notable depletion of synonymous variants [30]. Accordingly, at the nucleotide level, the adenine is conserved in all species with the exception of the claw frog where it is a thymidine (Figure 3). The Multiz Alignment of 100 Vertebrates track of the UCSC browser showed that the corresponding nucleotides in 66 of 91 vertebrate species is an adenine, while thymidine and cytosine are less abundant and none of the species have a guanine at the corresponding position (data not shown).

In the human transcript, the A-to-G transition does not result in an amino acid exchange. However, it is located at the fifth to last position of exon 8. It is known that consensus splice sites are not restricted to introns but extend into the exons, and in particular, the first and the last three exonic positions are an integral part of 3′ and 5′ splice site consensus sequences [25]. Hence, we aimed to analyze the effect of the c.750A > G variant on splicing in silico. Three splice site algorithms embedded in the Alamut visual software (v.2.12, Interactive Biosoftware, Rouen, France) predicted no effect on splicing (Supplementary Table S1). In contrast, the deep neural network SpliceAI tool [31] predicted a weakening of the exon 8 donor site with a Δscore of 0.45, which was slightly lower than the threshold of 0.5 (Supplementary Table S1).

The splicing process is not only determined by the consensus splice sites but requires auxiliary *cis*-elements in the exons known as exonic splicing enhancers (ESEs) [32] and exonic splicing silencers (ESSs) [33]. By binding to ESS and ESE sites, splicing regulatory proteins promote or antagonize splicing. HSF analysis (https://hsf.genomnis.com/home; accessed on 8 September 2021) predicted that the c.750A > G variant disrupted nine ESE sites and activated four ESS sites (Supplementary Table S2). The online tool EX-SKIP [34] revealed that the c.750A > G variant increased the predicted ESS/ESE ratio from 0.21 to 0.30, indicating a higher chance of exon skipping for the mutant allele (Supplementary Table S3). In addition, SpliceAid analysis [35] predicted that the c.750A > G variant abolished the binding site motifs for splicing factors HTra2beta1, SRp54, HTra2alpha, hnRNPH1, hnRNPH2, and SC35 and that it created binding motifs for splicing factors hnRNPA1 and SRp30c (Supplementary Table S4). Finally, the c.750A > G variant was predicted to increase exon 8 skipping by the HEXplorer algorithm (www2.hhu.de/rna/html/hexplorer_score.php, accessed on 15 February 2022) with a ΔHZ_EI_ value of −28.1 (Supplementary Figure S1).

### 2.3. In Vitro and In Vivo Splicing Assessments

**Figure 4:**
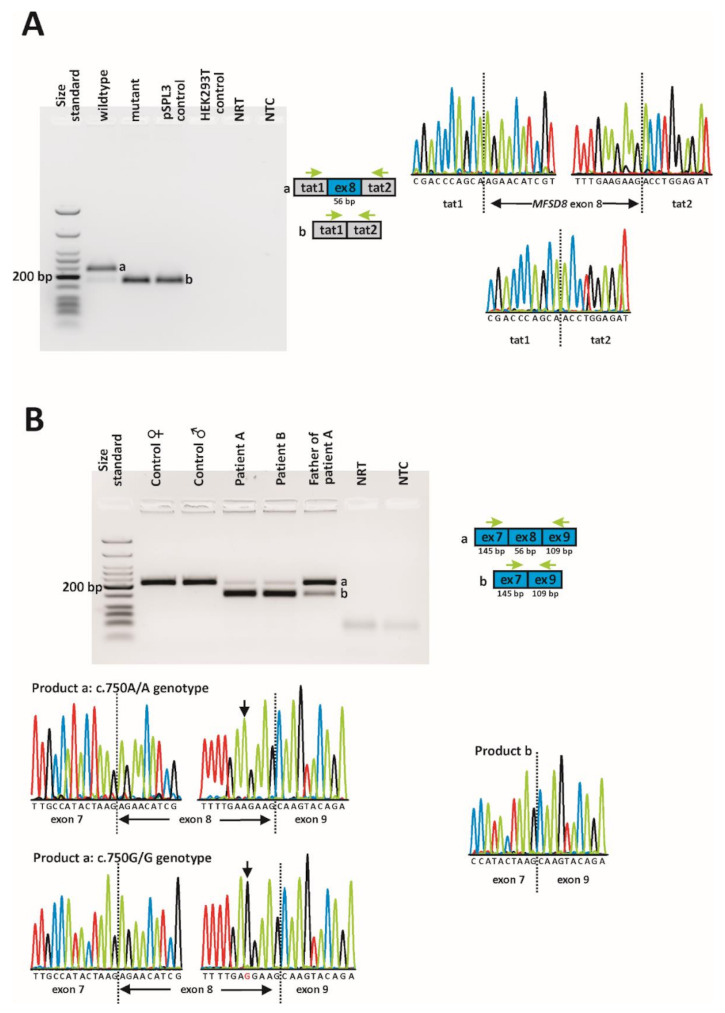
In vitro and in vivo splicing assessment of the c.750A > G variant. (**A**) Minigene assay. A full-size gel image of reverse transcription polymerase chain reaction (RT-PCR) products is shown on the left. Gel loading is as follows: A size standard (low molecular weight DNA ladder, NEB) was loaded in the leftmost lane. The RT-PCR products derived from HEK293T cells transfected with the wildtype minigene construct are shown in lane 2, while the RT-PCR product obtained upon transfection with the mutant minigene construct is shown in lane 3. RT-PCRs from the transfection with an empty pSPL3 vector (lane 4) and non-transfected HEK293T cells (lane 5) served as controls. NRT (lane 6), no reverse transcriptase control; NTC, no template control. Schemes of the amplified products and the sequence electropherograms are shown on the right side of the agarose gel picture. Grey boxes represent pSPL3 resident exons tat1 and tat2, and the blue box represents *MFSD8* exon 8. The green arrows indicate the locations of the RT-PCR primers. The expected sizes of the RT-PCR-amplified products were 230 bp in the event of normal splicing (product a) and 174 bp in the event of exon 8 skipping (product b). (**B**) Direct transcript analysis from blood samples. Following cDNA synthesis with random hexamers, RT-PCR was performed using primers located in *MFSD8* exon 7 and exon 9. A full-size gel image of the RT-PCR products is shown on the left. Lane 1: size standard (low-molecular-weight DNA ladder, NEB); lanes 2 + 3: RT-PCR from two healthy control subjects; lanes 4 + 5: RT-PCR from both patients; lane 6: RT-PCR from patient A’s father; lane 7: NRT; lane 8: NTC. Schemes of the amplified products are shown to the right of the agarose gel picture. Sequence electropherograms are shown below the gel image. The expected sizes of the RT-PCR-amplified products were 203 bp in the event of normal splicing (product a) and 147 bp in the event of exon 8 skipping (product b).

In order to assess the effect of the c.750A > G variant on splicing, we first made use of a minigene assay in human embryonic kidney (HEK) 293T cells. The reverse transcription polymerase chain reaction (RT-PCR) analysis of the RNA isolated from cells transfected with the wildtype minigene yielded a prominent band of the expected size (230 bp) corresponding to the inclusion of the *MFSD8* exon 8 between the vector resident exons tat1 and tat2 (Figure 4A). In addition, a smaller band with a much weaker intensity that corresponded to the skipping of exon 8 (174 bp) was observed. Sanger sequencing of subcloned RT-PCR products confirmed correct splicing of the major transcript and exon 8 skipping for the minor transcript. In contrast, transfection of cells with the mutant minigene resulted in a single transcript that was shown to lack exon 8. The transcript lacking exon 8 would lead, if translated, to a premature stop codon (p.(R233Sfs*5)). The out-of-frame skipping of exon 8 was confirmed in patient-derived cDNA obtained from blood lymphocytes (Figure 4B). Notably, a correctly spliced transcript was observed in both patients, although at much lower levels. Contrary to the minigene results, no endogenous exon 8 skipping was observed in the two control subjects.

## 3. Discussion

Genetic diagnostic testing based on genome sequencing revealed a novel, apparently synonymous substitution in exon 8 of the *MFSD8* gene in homozygous state in two members of a consanguineous Turkish family diagnosed with NCL. Four algorithms predicted that the c.750A > G variant causes an altered ratio of ESS/ESE binding motifs, indicating a higher chance of exon skipping for the mutant allele (Supplementary Tables S2–S4; Supplementary Figure S1). Hence, the mechanism that underlies the mis-splicing induced by the c.750A > G variant might be related to an altered balance of regulatory splicing factors. It is known that ESEs can compensate for the presence of “weak” (non-consensus) splice sites [36,37]. Although there are no consensus thresholds for “weak” splice sites, we would not consider the donor site of *MFSD8* exon 8 to be “weak” because the scores for the wildtype splice donor sequence are above the thresholds of three splicing algorithms (Supplementary Table S1). However, the fact that this rather small exon (56 bp) has a comparatively high number of ESEs is consistent with the observation that ESEs are significantly more frequent in weak exons than in strong exons [38]. In general, the bioinformatic analysis of ESEs and ESSs is often hampered by the assignment of the same sequence motif as ESE and ESS and the fact that their binding motifs are not unanimously characterized [39]. Further investigation is required to determine which splicing factor is directly involved in the mis-splicing promoted by the c.750A > G variant. However, using a minigene assay and transcript analysis of lymphocyte RNA, we demonstrated that the c.750A > G variant indeed promotes exon 8 skipping and is predicted to result in a truncated protein (p.(R233Sfs*5)).

The severity of this new variant appears to be comparable to other NCL-causing *MFSD8* variants, most of which have been associated with a syndromic NCL phenotype (https://www.ucl.ac.uk/ncl/cln7.shtml, accessed on 15 February 2022). While vision loss was previously described to occur within a narrow age range in juvenile NCL patients, the onset and progression of other symptoms such as intellectual disability, language disability, motor function, and seizures were shown to be more variable [40]. Both inter- and intrafamilial phenotypic variability for the same genotype have been described for different forms of NCLs [41,42,43], especially with respect to the age at the disease onset, which often differs by 2–5 years in siblings. Few reports described a more pronounced intrafamilial variability. Järvelä and colleagues described two sisters who were homozygous for a recurrent deletion in the *CLN3* gene, became blind at the ages of 11 and 20, respectively and were wheelchair-bound at 15 and 23, respectively [44]. Wisniewski and colleagues described three siblings with mutations in the *CLN2* gene: The onset in seizures varied from nine to 20 years and visual impairment developed only in one sibling [45]. For the *MFSD8* gene, the same genotype has been shown to cause both NCL and nonsyndromic retinopathy, but only in unrelated patients [22]. In our study, we have observed a marked intrafamilial disease variability. While patient A presented a late infantile onset, intellectual disability, and a considerable cerebellar ataxia syndrome, patient B presented an adult onset, no intellectual disability, and only a mild atactic syndrome (Table 1). The difference in the onset of first neurologic symptoms was 15 years and that of ophthalmologic symptoms was 12 years. The cMRI showed atrophy of the cerebellum in both patients, whereas only patient A showed cerebral atrophy.

Electron microscopy was performed on skin biopsies of both subjects. The male patient’s biopsy lacked an accumulation of lipopigment. This is in accordance with previous findings, since the accumulation of specific lipopigments is not always detectable in extracerebral tissues [1,46]. The female patient’s biopsy showed multiple vacuoled sweat gland epithelial cells. The ultrastructure of these vacuoles revealed regular lipofuscin and no disease-specific structures such as fingerprint or curvilinear bodies. Regular lipofuscin may accumulate in adult NCL along with specific inclusion or in the absence of these [47].

In general, the ophthalmologic, neurologic and MRI findings observed in our subjects are consistent with the previously described features of NCL [3]. Currently, we have no explanation for the marked intrafamilial phenotypic variability. Since we have performed genome sequencing in both patients, we can rule out *cis*-acting variants in the vicinity of *MFSD8* that might influence expression levels. Whether the variable expressivity in NCL is caused by modifier genes, environmental or lifestyle factors remains to be determined.

Transcript analysis of the *MFSD8* gene (i.e., establishing the effect of variants on splicing) is hampered by the presence of various isoforms. The longest transcript (NM_152778.3) comprises 13 exons and is considered the main isoform. In addition to the main transcript, small amounts of *MFSD8* transcripts lacking one or two exons have been observed in blood lymphocytes from healthy control subjects, for instance transcripts lacking exon 7, exon 8, or both exons 7 and 8 [8] or transcripts lacking exon 11 [18]. While those transcripts lacking either exon 7, exon 8, or exon 11 are out of frame and are predicted to be subjected to nonsense-mediated mRNA decay (NMD), the transcript lacking both exon 7 and exon 8 is predicted to result in an in-frame deletion of 67 amino acids. The functional relevance of these less abundant transcripts is unknown. Of note, variants in exons 7, 8, and 11 or at their intron boundaries have been repeatedly identified as disease-causing in NCL patients, indicating that these exons are functionally relevant. To date, the HGMD database lists three variants at the splice donor site of exon 8. For two variants, namely c.754 + 1G > A and c.754 + 1G > T, no mRNA analysis has been performed [3,48], but Siintola and colleagues performed direct mRNA analysis for the c.754 + 2T > A variant [8]. In their study, a control subject showed a prominent transcript compatible with correct splicing, but also minor transcripts compatible with transcripts lacking either exon 7, exon 8, or both exon 7 and exon 8 [8]. In comparison with the control subject, a patient diagnosed with CLN and homozygous for the c.754 + 2T > A variant showed an almost complete lack of the correctly spliced transcript, while an increased expression of transcripts lacking exon 8 and one lacking both exon 7 and exon 8 was seen [8]. To reduce the complexity of transcripts, we have performed RT-PCR using a forward primer located in exon 7 and a reverse primer located in exon 9. In this way, we did not capture endogenous transcripts lacking exon 7 or exon 11. In contrast to the study of Siintola and colleagues, we could not observe endogenous exon 8 skipping in controls [8]. This is likely due to different primers and PCR conditions. While the two control subjects in our study expressed only the correctly spliced transcript, the two affected subjects expressed mainly a transcript lacking exon 8 and only small amounts of the correctly spliced transcript. Of note, the father of proband A, who has been shown to be heterozygous for the c.750A > G variant, expressed proportionately more of the correctly spliced transcript, which may indicate that the mutant transcript is partially degraded by NMD. However, RT-PCR was performed as an end-point analysis, so any interpretation of the quantities should be taken with caution.

The skipping of exon 8 is predicted to cause a frameshift and premature termination codon (PTC). Hence, the mutant transcript is predicted to be subjected to NMD [49]. Even if the mutant transcript were transcribed, it would result in a truncated protein. HGMD lists at least 10 different variants that are predicted to result in PTCs downstream of the c.750A > G variant. These variants are thought to result in no or residual protein function. Hence, we assume a similar effect for the c.750A > G variant.

## 4. Materials and Methods

### 4.1. Subjects

The two subjects described in this study were recruited and clinically examined at the Eye Hospital and at the Department of Neurology and Neuroscience, University of Tübingen, Germany. Patient A is a 20-year-old male, and patient B is a 26-year-old female. Both subjects are from a consanguineous Turkish family but did not provide consent to disclose their exact relationships. Legal consent was obtained for this study, which was approved by the institutional ethical committee (349/2003V and 116/2015BO2).

### 4.2. Clinical Assessment

Both subjects underwent several follow-ups with multimodal ophthalmologic examinations. The clinical examination included a detailed medical history, BCVA testing, slit-lamp examination with funduscopy in mydriasis, static or/and kinetic perimetry (Octopus 900; Haag-Streit International, Wedel, Germany), OCT (Heidelberg Engineering, Heidelberg, Germany), fundus photography and wide-field FAF (California Optos, Dunfermline, UK), color vision testing using Lanthony and Farnsworth panel tests (OCULUS, Wetzlar, Germany), full-field electroretinography (ERG) according to the International Society for Clinical Electrophysiology of Vision (ISCEV) standards with an Espion E2/E3 system (Diagnosys LLC, Cambridge, UK), and dark-adapted full-field scotopic threshold (FST) with blue and red light (Diagnosys LLC, Cambridge, UK) where 0 dB was set to 0.01 cd·s/m^2^. All ophthalmologic examinations except for the FST were performed in both eyes.

A comprehensive clinical neurological examination was performed by a board neurologist specialized on complex rare neurogenetic diseases (L.Z.) at the Centre for Rare Diseases, University of Tübingen. The severity of ataxia was rated using the SARA [50]. cMRI was recorded externally and revisited with a special focus on brain atrophy and white matter changes.

### 4.3. Genetic Diagnostic Testing

EDTA blood samples were obtained from both probands. DNA was isolated from peripheral blood leukocytes using standard procedures. Genetic diagnostic testing was performed by genome sequencing. Briefly, sequencing (2 × 150 bp paired-end reads) was performed on an Illumina platform (NovaSeq6000). Methodological details have already been published [51].

### 4.4. Bioinformatic Analysis

Nucleotide conservation among vertebrates of the human *MFSD8* c.750 A nucleotide was assessed using the Multiz Alignment of 100 Vertebrates track of the University of California Santa Cruz (UCSC) Genome Browser-hg19 assembly tool [52]. The prediction of splicing alterations was performed with the Alamut visual software using default settings (v.2.12, Interactive Biosoftware, Rouen, France) and the SpliceAI [31] lookup tool from the Broad Institute (https://spliceailookup.broadinstitute.org/, accessed on 15 February 2022). The consequences on the splicing enhancer and silencer target motifs were analyzed with the online tools HSF (https://hsf.genomnis.com/home, accessed on 15 February 2022), EX-SKIP [34], SpliceAid [35], and the ΔHZ_EI_ value provided by the HEXplorer algorithm (www2.hhu.de/rna/html/hexplorer_score.php, accessed on 15 February 2022) using an exon inclusion threshold of −20.

Variant nomenclature in this study is in accordance with Human Genome Variation Society recommendations [53] and based on GenBank accession numbers NM_152778.3. and NP_689991.1.

### 4.5. Minigene Assays

Minigene assays were performed as described previously [54]. Briefly, a genomic segment of the *MFSD8* gene (GrCh37/hg19 4: 128,859,246–128,860,517; corresponding to exon 8 and flanking intronic sequences) was amplified from patient genomic DNA using a proofreading polymerase and cloned into the pSPL3 minigene plasmid vector. After verifying the integrity of the minigene, the plasmid was then used to introduce the wildtype allele by site-directed mutagenesis [55]. The resulting minigene constructs in their wildtype and mutant versions were used to transfect HEK293T/17 cells (ATCC^®^ CRL-11268^TM^), which were then analyzed with respect to the splicing of minigene-derived transcripts using RT-PCR. The subcloning of RT-PCR products was performed using the CloneJET PCR Cloning Kit (Thermo Fisher Scientific, Dreieich, Germany) according to the manufacturer’s instructions.

### 4.6. Direct mRNA Analysis from Blood Cells

Venous whole blood was collected in S-Monovette^®^ RNA Exact tubes (Sarstedt, Nümbrecht, Germany). RNA isolation was performed using the NucleoSpin^®^ RNA Blood Midi kit (Macherey-Nagel, Düren, Germany) according to the manufacturer’s protocol. Four hundred nanograms of total RNA were used for cDNA synthesis using random hexamers and the Maxima H Minus Reverse Transcriptase Kit according to the manufacturer’s protocol (Thermo Fisher Scientific, Dreieich, Germany). RT-PCR was performed using 2 µL cDNA, a forward primer located in exon 7 (5′-AAGGTGTGACATGGGATGTG-3′), and a reverse primer located in exon 9 (5′-ATTTCCTTGGGGAACCTGAG-3′).

## 5. Conclusions

To conclude, we identified a synonymous substitution in *MFSD8* in two members of a consanguineous Turkish family diagnosed with NCL, demonstrating marked intrafamilial phenotypic variability. Assessing the pathogenicity of a variant that is silent at the translational level is challenging, even if it is in a known disease gene. Several lines of evidence support our assumption that the c.750A > G variant is the underlying genetic cause of disease in our study. First, despite the marked intrafamilial phenotypic variability observed between the two subjects, their phenotypes fell entirely within the range of reported CLN7-associated mutations. Second, the c.750A > G variant was the only putative pathogenic variant prioritized in NCL-associated genes identified by the genome diagnostic sequencing of both subjects. Third, the variant is absent from large population databases which argues against the possibility of being a rare benign variant. Fourth, the bioinformatic analyses predicted a higher probability of the mutant allele to cause exon skipping. Finally, both in vitro and in vivo studies confirmed that the variant caused an out-of-frame skipping of exon 8. Variants at the donor site of exon 8 have been repeatedly identified as disease-causing in NCL patients, indicating that this exon is functionally relevant.

Our study increases the spectrum of disorders in which exonic synonymous substitutions exert pathogenic splicing defects. In addition, the knowledge of the molecular disease mechanism of the underlying variant is crucial for the development of tailored gene correction approaches.

## Figures and Tables

**Table 1:** Summary of phenotypic features.

Main Feature	Detailed Characteristics	Patient A	Patient B
Medical history	Gender	Male	Female
	First neurological symptom	Speech delay	Epileptic seizure
	First ophthalmological symptom	Blurred vision	Blurred vision
	Age at the first neurological symptom	4 years	19 years
	Age at the first ophthalmological symptom	10 years	22 years
	Age at the last visit	20 years	26 years
Neurological symptoms	Delayed speech	+	-
	Intellectual disability	+	-
	Psychomotor degeneration	+	-
	Visual hallucinations	-	-
	Aphasia	-	-
	Dysarthria	+	-
	Seizures	+	+
	Ataxia of extremities	+	+
	Gait ataxia	+	+
	Saccadic gaze	+	+
	Myoclonus	-	+
Vision problems	Photophobia	+	+
	Nystagmus	+	-
	Loss of visual acuity	+	+
	Foveal thinning of the retinal layer	+	+
	Diminished or absent ERG responses	n/a	+
cMRI	Cerebellar atrophy	+	+
	Cerebral atrophy	+	-
	White matter changes	-	-
	Other abnormalities	-	-

ERG, electroretinography; cMRI, cerebral magnetic resonance imaging; n/a, not analyzed.

## Data Availability

All data are contained within the article or Supplementary materials.

## Supplementary Materials

The following supporting information can be downloaded at: https://www.mdpi.com/article/10.3390/ijms23042271/s1.
