# Supplementary material for: A Novel, Apparently Silent Variant in MFSD8 Causes Neuronal Ceroid Lipofuscinosis with Marked Intrafamilial Variability
Source: Int J Mol Sci. 2022 Feb 18;23(4):2271. doi: 10.3390/ijms23042271 (PMC8877174; doi:10.3390/ijms23042271)
Supplement: Supplementary file 1 [file ijms-23-02271-s001.zip › ijms-1589060-supplementary.pdf]

**Supplementary Table S1:** *In silico* splice site predictions from four algorithms for the c.750A>G variant				
Program	NNSplice	MaxEntScan	SSF-like	SpliceAI*
Score range	[0-1]	[0-12]	[0-100]	[0-1]
Threshold	≥0.4	≥0	≥70	≥0.5
Score	0.9	8.4	76.5	0.45
Score change**	(± 0 %)	(± 0 %)	(± 0 %)	
*SpliceAI prediction is given as Δscore (difference between reference and mutant score); **compared with wildtype.				

**Supplementary Table S2:** Results of HSF disclosing the effect of the c.750A>G variant on splice enhancers and silencers		
**Signal**	**Interpretation**	
**Alteration of auxiliary sequences**	**Significant alteration of ESE / ESS motifs ratio (-10)**	
Name	Position	Sequence
RESCUE ESE (ESE Site Broken)	chr4:127938787	AGAAGG
EIE (ESE Site Broken)	chr4:127938787	AGAAGG
ESE_Tra2 (ESE Site Broken)	chr4:127938788	AAGAA
ESS_hnRNPA1 (New ESS Site)	chr4:127938789	GAGGAA
Sironi_motif1 (ESS Site Broken)	chr4:127938789	GAAGAAGG
PESE (ESE Site Broken)	chr4:127938789	GAAGAAGG
ESE_SRp55 (New ESE Site)	chr4:127938790	TGAGGA
RESCUE ESE (ESE Site Broken)	chr4:127938790	TGAAGA
Sironi_motif2 (New ESS Site)	chr4:127938790	TGAGGAA
PESE (ESE Site Broken)	chr4:127938790	TGAAGAAG
PESE (New ESE Site)	chr4:127938790	TGAGGAAG
RESCUE ESE (ESE Site Broken)	chr4:127938791	TTGAAG
PESE (ESE Site Broken)	chr4:127938791	TTGAAGAA
EIE (ESE Site Broken)	chr4:127938792	TTTGAA
Sironi_motif2 (New ESS Site)	chr4:127938792	TTTGAGG
Sironi_motif1 (New ESS Site)	chr4:127938792	TTTGAGGA

**Supplementary Table S3:** EX-SKIP comparison of the predicted numbers of ESSs and ESEs identified within the junction between *MFSD8* exon 8-intron 8 and their ratio for both reference and mutant sequences																
**Seq**	**PESS (count)**	**FAS-ESS hex2 (count)**	**FAS-ESS hex3 (count)**	**IIE (count)**	**IIE (sum)**	**NI-ESS trusted (count)**	**NI-ESS all (sum)**	**PESE (count)**	**RESCUE-ESE (count)**	**EIE (count)**	**EIE (sum)**	**NI-ESE trusted (count)**	**NI-ESE all (sum)**	**ESS (total)**	**ESE (total)**	**ESS/ESE (ratio)**
WT	0	0	0	3	54.7993	0	-0.6639	2	4	4	106.1529	4	4.6351	3	14	**0.21**
MUT	0	0	0	3	58.4170	0	-0.6639	1	2	3	31.3720	4	4.2449	3	10	**0.30**
Verdict: The mutant allele has a higher chance of exon skipping than the wildtype allele.																

**Supplementary Table S4:** SpliceAid analysis of RNA target motifs bound by splicing proteins in the reference or c.750A>G *MFSD8* sequences using the junction between exon 8-intron 8 as an input									
Binding sites present ONLY in the first (WILD-TYPE) sequence									
**Position**	**Protein Name**	**Recognized Sequence**	**Score**	**Protein Notes**	**PubMed ID**	**Reference**	**Gene/Construct (Target RNA)**	**Splicing Assay**	**Binding Assay**
71-76	HTra2beta1	GAAGAA	10	Gene Name and Synonymous: SFRS10, splicing factor arginine/serine-rich 10 (transformer 2 homolog, Drosophila), TRA2B, SFRS10, TRAN2B, TRA2-BETA, Htra2-beta, DKFZp686F18120.	20926394	Tsuda K, Someya T, Kuwasako K, Takahashi M, He F, Unzai S, Inoue M, Harada T, Watanabe S, Terada T, Kobayashi N, Shirouzu M, Kigawa T, Tanaka A, Sugano S, Güntert P, Yokoyama S, Muto Y. (2010) Structural basis for the dual RNArecognition modes of human Tra2-ß RRM. Nucleic Acids Res. 39(4):1538-1553.	Synthesized sequences		NMR spectroscopy
72-76	HTra2beta1	AAGAA	7	Gene Name and Synonymous: SFRS10, splicing factor arginine/serine-rich 10 (transformer 2 homolog, Drosophila), TRA2B, SFRS10, TRAN2B, TRA2-BETA, Htra2-beta, DKFZp686F18120.	9546399	Tacke R, Tohyama M, Ogawa S, Manley JL. (1998) Human Tra2 proteins are sequence-specific activators of premRNA splicing. Cell. 93(1): 139-148.	Sequences of 20 nt random for SELEX. Sequence of beta-globin [3043] and constructs of murine IgM-based premRNA for in vitro splicing.	In vitro splicing in HeLa S100 and nuclear extracts.	SELEX of 20nt random with recombinant protein, confirmed by EMSA in HeLa nuclear extract and S100. EMSA with recombinant protein.
72-77	SRp54	AAGAAG	-5	Gene Name and Synonymous: SFRS11, splicing factor, arginine/serine-rich 11, p54.	16943417	Wu JY, Kar A, Kuo D, Yu B, Havlioglu N. (2006) SRp54 (SFRS11), a regulator for tau exon 10 alternative splicing identified by an expression cloning strategy. Mol Cell Biol. 26(18):6739-6747.	Construct of Tau MAPT [4137] EX9 - INT9 - EX10 - INT10 - EX11 with GFP cDNA inserted into EX11.	In vivo splicing in HEK293.	Positive clones identified by fluorescenceactivated cell sorting and visual inspection. Confirmed by UV crosslink, immunoprecipitation, SDS-PAGE.
72-77	HTra2beta1	HTra2beta1 AAGAAG	5	Gene Name and Synonymous: SFRS10, splicing factor arginine/serine-rich 10 (transformer 2 homolog, Drosophila), TRA2B, SFRS10, TRAN2B, TRA2-BETA, Htra2-beta, DKFZp686F18120.	16943417	Wu JY, Kar A, Kuo D, Yu B, Havlioglu N. (2006) SRp54 (SFRS11), a regulator for tau exon 10 alternative splicing identified by an expression cloning strategy. Mol Cell Biol. 26(18):6739-6747.	Construct of Tau MAPT [4137] EX9 - INT9 - EX10 - INT10 - EX11 with GFP cDNA inserted into EX11.	In vivo splicing in HEK293.	Positive clones identified by fluorescenceactivated cell sorting and visual inspection. Confirmed by UV crosslink, immunoprecipitation, SDS-PAGE.
72-76	HTra2alpha	AAGAA	7	Gene Name and Synonymous: TRA2A, transformer-2 alpha, HSU53209.	9546399	Tacke R, Tohyama M, Ogawa S, Manley JL. (1998) Human Tra2 proteins are sequence-specific activators of premRNA splicing. Cell. 93(1): 139-148.	Sequences of 20 nt random for SELEX. Sequence of beta-globin [3043] and constructs of murine IgM-based premRNA for in vitro splicing.	In vitro splicing in HeLa S100 and nuclear extracts.	SELEX of 20nt random with recombinant protein, confirmed by EMSA in HeLa nuclear extract and S100. EMSA with recombinant protein.
72-76	hnRNP H1	AAGAA	-2	Gene Name and Synonymous: HNRNPH1, heterogeneous nuclear ribonucleoprotein H1 (H), HNRPH, HNRPH1, hnRNPH, DKFZp686A15170.	17337441	Schaub MC, Lopez SR, Caputi M. (2007) Members of the heterogeneous nuclear ribonucleoprotein H family activate splicing of an HIV-1 splicing substrate by promoting formation of ATP-dependent spliceosomal complexes. J Biol Chem. 282(18):13617-13626.	Synthesized sequences		RNA-affinity chromatography assays with HeLa NE and immunoblotting or with recombinant protein.
72-76	hnRNP H2	AAGAA	-2	Gene Name and Synonymous: HNRNPH2, heterogeneous nuclear	17337441	Schaub MC, Lopez SR, Caputi M. (2007) Members of the heterogeneous nuclear	Synthesized sequences		RNA-affinity chromatography assays with HeLa NE and immunoblotting or with recombinant

				ribonucleoprotein H2 (H'), FTP3, HNRPH', HNRPH2, hnRNPH'.		ribonucleoprotein H family activate splicing of an HIV-1 splicing substrate by promoting formation of ATP-dependent spliceosomal complexes. J Biol Chem. 282(18):13617-13626.			protein.
73-77	SC35	AGAAG	7	Gene Name and Synonymous: SFRS2, splicing factor arginine/serine-rich 2, SC-35, SFRS2A, SRp30b, PR264. SC35 accelerates transcriptional elongation (cotranscriptional splicing) (PMID: 18641664).	11847131	Caputi M, Zahler AM. (2002) SR proteins and hnRNP H regulate the splicing of the HIV-1 tev-specific exon 6D. EMBO J. 21(4): 845-855.	Construct of HIV-1 env [155971] EX_6D and part of flanking introns.	In vitro splicing with HeLa nuclear extracts	RNA affinity chromatography assay and immunoblot.

Binding sites present ONLY in the second (MUTATED) sequence

Position	Protein Name	Recognized Sequence	Score	Protein Notes	PubMed ID	Reference	Gene/Construct (Target RNA)	Splicing Assay	Binding Assay
71-77	hnRNP A1	GAGGAAG	5	Gene Name and Synonymous: HNRNPA1, heterogeneous nuclear ribonucleoprotein A1, HNRPA1, MGC102835. hnRNP A1 carries bidirectional shuttling signals that serve for both nuclear localization and export (PMID:8521471).	23430061	Oh HK, Lee E, Jang HN, Lee J, Moon H, Sheng Z, Jun Y, Loh TJ, Cho S, Zhou J, Green MR, Zheng X, Shen H. (2013) hnRNP A1 contacts exon 5 to promote exon 6 inclusion of apoptotic Fas gene. Apoptosis. 18(7):825-835.	Constructs of wt or mutated FAS [355] EX5 - INT5 - EX6 - INT6 - EX7. Synthesized sequences.	In vivo splicing in HeLa, HCT-116, MDAMB-231.	In vivo splicing using wt and mutated sequences in MDA-MB-231, HeLa, HCT-116
72-76	SRp30c	AGGAA	2	Gene Name and Synonymous: SFRS9, splicing factor arginine/serine-rich 9.	17548433	Paradis C, Cloutier P, Shkreta L, Toutant J, Klarskov K, Chabot B. (2007) hnRNP I/PTB can antagonize the splicing repressor activity of SRp30c. RNA 13: 1287-1300.	Synthesized oligos. Sequences of 20nt random for SELEX.	In vitro splicing with HeLa nuclear extracts, siRNA.	SELEX of 20nt random with recombinant protein. EMSA, UV crosslink, SDS-PAGE with HeLa nuclear extracts.

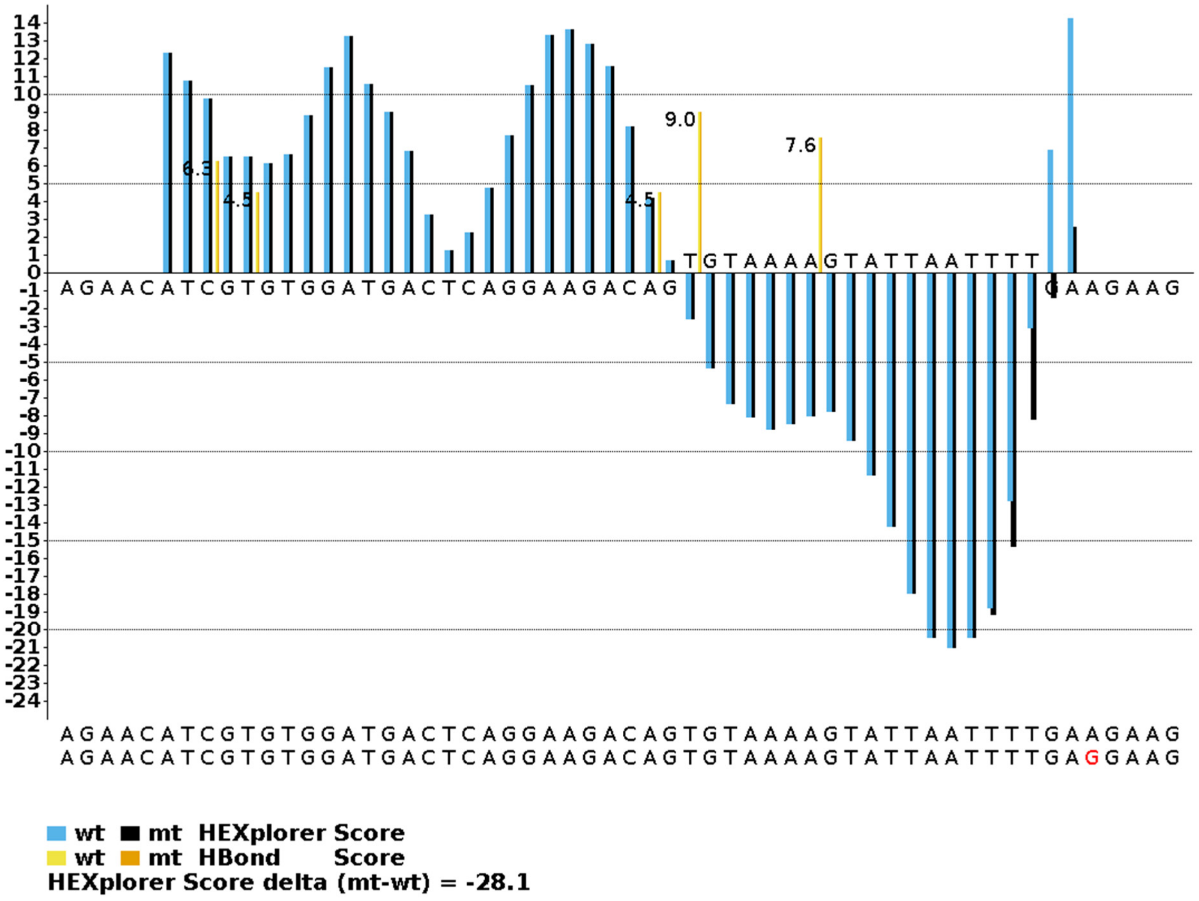

Supplementary Figure S1: Graphical presentation of data extracted from the HEXplorer algorithm for the wild type and mutated sequences of *MFSD8* exon 8 (c.699_754).
